# Midlife blood pressure is associated with the severity of white matter hyperintensities: analysis of the UK Biobank cohort study

**DOI:** 10.1093/eurheartj/ehaa756

**Published:** 2020-11-26

**Authors:** Karolina Agnieszka Wartolowska, Alastair John Stewart Webb

**Affiliations:** Wolfson Centre for Prevention of Stroke and Dementia, Nuffield Department of Clinical Neurosciences, University of Oxford, John Radcliffe Hospital, Headley Way, Oxford OX3 9DU, UK; Wolfson Centre for Prevention of Stroke and Dementia, Nuffield Department of Clinical Neurosciences, University of Oxford, John Radcliffe Hospital, Headley Way, Oxford OX3 9DU, UK

**Keywords:** Small vessel disease, Diastolic blood pressure, Hypertension, White matter hyperintensities, Magnetic resonance imaging

## Abstract

**Aims:**

White matter hyperintensities (WMH) progress with age and hypertension, but the key period of exposure to elevated blood pressure (BP), and the relative role of systolic BP (SBP) vs. diastolic BP (DBP), remains unclear. This study aims to determine the relationship between WMH and concurrent vs. past BP.

**Methods and results:**

UK Biobank is a prospective community-based cohort of 40–69-year olds from 22 centres, with magnetic resonance imaging in a subgroup of over 40 000 people at 4–12 years after baseline assessment. Standardized associations between WMH load (WMH volume normalized by total white matter volume and logit-transformed) and concurrent vs. past BP were determined using linear models, adjusted for age, sex, cardiovascular risk factors, BP source, assessment centre, and time since baseline. Associations adjusted for regression dilution bias were determined between median WMH and usual SBP or DBP, stratified by age and baseline BP.

In 37 041 eligible participants with WMH data and BP measures, WMH were more strongly associated with concurrent SBP [DBP: β = 0.064, 95% confidence interval (CI) 0.050–0.078; SBP: β = 0.076, 95% CI 0.062–0.090], but the strongest association was for past DBP (DBP: β = 0.087, 95% CI 0.064–0.109; SBP: β = 0.045, 95% CI 0.022–0.069), particularly under the age of 50 (DBP: β = 0.103, 95% CI 0.055–0.152; SBP: β = 0.012, 95% CI −0.044 to 0.069). Due to the higher prevalence of elevated SBP, median WMH increased 1.126 (95% CI 1.107–1.146) per 10 mmHg usual SBP and 1.106 (95% CI 1.090–1.122) per 5 mmHg usual DBP, whilst the population attributable fraction of WMH in the top decile was greater for elevated SBP (19.1% for concurrent SBP; 24.4% for past SBP). Any increase in BP, even below 140 for SBP and below 90 mmHg for DBP, and especially if requiring antihypertensive medication, was associated with increased WMH.

**Conclusions:**

WMH were strongly associated with concurrent and past elevated BP with the population burden of severe WMH greatest for SBP. However, before the age of 50, DBP was more strongly associated with WMH. Long-term prevention of WMH may require control of even mildly elevated midlife DBP.

**See page 758 for the editorial comment on this article (doi: 10.1093/eurheartj/ehaa971)**

## Introduction

White matter hyperintensities (WMH) are associated with an increased risk of stroke,^1^ ^,^ ^2^ dementia,^1–3^ refractory depression,^4^ and functional decline in older age.^4^ They are present in >50% of patients over the age of 65 and almost everyone over the age of 80.^5^ However, the underlying pathophysiological mechanisms are still unclear^2^ despite evidence for a strong genetic component.^6^ Hypertension (HT) is the single strongest treatable risk factor,^2^ ^,^ ^7–9^and despite evidence from post hoc analyses of randomized controlled trials suggesting that antihypertensive treatment reduces the progression of WMH,^10^ there is a lack of conclusive evidence that lowering blood pressure (BP) reduces the risk of stroke and dementia or which patient groups are most likely to benefit from this treatment. In particular, the key period of exposure to elevated BP, and, therefore, the optimal treatment period, is unknown. Concurrent associations with arterial stiffness,^11^ endothelial dysfunction,^2^ impaired cerebrovascular reactivity, and cerebral pulsatility^11^ ^,^ ^12^ suggest the importance of long-term BP changes, but the longitudinal interactions between WMH, age, HT, and the pulsatile [systolic BP (SBP)] vs. the steady-state [diastolic BP (DBP)] component of BP are unclear.^11^

Elevated BP has been positively associated with concurrent WMH volume in a number of studies^13^ ^,^ ^14^ but with a weaker effect for concurrent BP than for measurements taken 3 years,^12^ 7 years,^15^ or even 10–20 years^8^ previously, with the more distant measurements having a stronger effect.^8^ Some studies have reported that both high SBP and DBP are positively associated with WMH volume later in life^12^ ^,^ ^13^ but other studies have described these associations for DBP but not for SBP.^14^ However, these studies have analysed small- to moderate-sized populations, at different ages, different BP levels, and different durations of follow-up, limiting the ability to determine the role of midlife BP levels on mid- and late-life WMH.

This study aims to use UK Biobank, a very large community-based cohort study, to determine the strength of associations between WMH and concurrent vs. past BP, and the relative strength of these associations stratified by age.

## Methods

### Study design, settings, and participants

UK Biobank is a community-based cohort recruited from the general, midlife population aged between 40 and 69 years. The baseline demographic, physiological, and clinical data were collected between March 2006 and October 2010 and the follow-up data and magnetic resonance imaging (MRI) data were collected between August 2014 and October 2019. Participants eligible for cross-sectional analyses had non-missing values for SBP and DBP and reported volume of WMH, as derived from structural MRI by the UK Biobank core imaging team. Participants included in the longitudinal analyses also had non-missing BP measures at baseline. People with conditions confounding the assessment of WMH volume were excluded. These conditions, such as multiple sclerosis or other demyelinating disorders, cerebral infarction, encephalitis or brain abscess, brain tumour, or systemic lupus erythematosus, were identified using the International Statistical Classification of Diseases and Related Health Problems 10th Revision (ICD-10) codes and the self-reported cerebrovascular diseases field.

Ethical approval, consenting of participants, and data collection have been done by the UK Biobank team under ethical approval from the North West Multi-Centre Research Ethical Committee (MREC). UK Biobank data are openly accessible following a direct application by valid researchers. This research has been conducted using the UK Biobank Resource under application number 41213.

### Data sources and management

The primary outcome was WMH load, defined as the proportion of total white matter volume classified as WMH on T1-weighted and Fluid-attenuated Inversion Recovery (FLAIR) MRI scans using BIANCA.^16^ ^,^ ^17^ WMH load was logit-transformed to normalize and stabilize the variance. Sensitivity analyses were performed with WMH load as a proportion of the total brain volume calculated by adding the total, unscaled, volume of grey matter, white matter, and cerebrospinal fluid.^18^

In the UK Biobank, BP was measured twice by trained nurses after the participant had been at rest for at least 5 min in the seated position with a digital sphygmomanometer (Omron 705 IT; OMRON Healthcare Europe B.V., Hoofddorp, Netherlands) with a suitably sized cuff. Individual measures of SBP and DBP were averaged within a visit. Automated BP measurements were used as the preferred source of BP data, if automated BP was missing, then manual BP was used, and when it was not available, a BP from the pulse wave analysis (PWA) was used. Sensitivity analyses excluding PWA-based BP were performed. In this study, the hypertensive status of participants was defined according to standard BP thresholds, with the following cut-offs (with left-closed intervals): DBP <70, 70–80, 80–90, ≥90 mmHg and SBP <120, 120–130, 130–140, ≥140 mmHg.^19^ ^,^ ^20^

Arterial stiffness index (ASI) was measured in the UK Biobank using the PulseTrace PCA2 (CareFusion, San Diego, CA, USA), to record a 10–15-s pulse waveform by finger photoplethysmography, which was repeated on a larger finger or the thumb if less than two-thirds of the waveform were visible or if the waveform did not stabilize within 1 min. ASI was derived from the interval between the forward and presumed aortic-reflected reverse-travelling pulse wave, standardized to the standing height.

Extracted demographic and clinical characteristics included: age, sex and self-reported cardiovascular risk factors including obesity, hypercholesterolaemia, HT, diabetes, smoking status, and diagnoses of HT or cerebrovascular diseases. The number of included participants, as well as data quality and completeness, depended on the quality and completeness of the UK Biobank dataset.

### Statistical analysis

#### Relationships between blood pressure and white matter hyperintensities

Cross-sectional and longitudinal relationships between BP and WMH load were determined by linear models, unadjusted, adjusted for age and sex, and fully adjusted for both SBP and DBP, age, sex, ASI, and cardiovascular risk factors, including smoking and diagnosis of diabetes, source of BP data, and assessment centre. Longitudinal analyses were additionally adjusted for the time difference between the baseline and follow-up assessment. Results were presented as standardized coefficients and per 10 mmHg SBP and 5 mmHg DBP. Interactions between SBP or DBP and gender were determined in fully adjusted cross-sectional and longitudinal models.

Fully adjusted analyses were stratified by age groups (with left-closed intervals): <50, 50–60, and ≥60 years for the baseline data and <60, 60–70, and ≥70 years for the follow-up data. Stratified analyses also were repeated for men and women separately and for short and long follow-up duration (median split). Results of these analyses were presented as forest plots and tables.

Due to a strong positive skew of the distribution of WMH, the population burden of patients with high WMH load due to elevated SBP and DBP was determined as the population attributable fraction (PAF). PAF was estimated as a fraction of people in the top decile of WMH load due to SBP over 120 mmHg or DBP over 70 mmHg.

#### Relationships between usual blood pressure and white matter hyperintensities

To determine interactions between usual BP, age, WMH, and the threshold definition of HT, the population was stratified by age and BP, adjusted for regression dilution bias.^11^ Median WMH load within each stratum was determined relative to the median value in the reference group (normotensive people in the youngest age group). Usual BP was determined by the mean BP at follow-up for participants stratified by baseline BP level at the pre-defined thresholds. The magnitude of the association between mean BP and change in relative median WMH load per group was estimated by linear regression of usual SBP and DBP with the log of the ratio between the median WMH within each group and the reference group.^21^ Confidence intervals were calculated using non-parametric bootstrapping.

#### Relationships between white matter hyperintensities, age, hypertension, and antihypertensive medication

The effect of the BP and antihypertensive medication on WMH was analysed by stratifying participants according to their age, BP level, and whether they were on antihypertensive treatment. Participants with SBP <120 mmHg and DBP <70 mmHg were defined as low-range normotensive or very well-controlled hypertensive; those with SBP 120–130 mmHg or DBP 70–80 mmHg as high-range normotensive or well-controlled hypertensive, SBP 130–140 mmHg or DBP 80–90 mmHg as pre-hypertensive or controlled hypertensive; and those with SBP ≥140 mmHg or DBP ≥90 mmHg as untreated hypertensive or uncontrolled hypertensive.^19^ ^,^ ^20^

The median logit-transformed WMH load, within each subgroup, was presented as boxplots with median and interquartile range and with the underlying data distribution represented as violin plots. Differences in logit-transformed WMH load between the unmedicated people in the lowest BP group and other categories were formally tested using a multivariable linear model adjusted for a decade of age, sex, ASI, smoking status, diagnosis of diabetes, assessment centre, and the time between visits (in the longitudinal analysis).

### Statistical software and packages 

All data management and analyses were performed using R version 4.0.0 using the *data.table* package version 1.12.8, the *magrittr* package version 1.5, and the *lme4* package version 1.1-21. Figures were plotted using *ggplot2* version 3.2.1 and annotated using *captioner* version 2.2.3.9000. In addition, *stringr* version 1.4.0 and *fst* 0.9.0 packages were used for data management. The manuscript was typeset using *knitr* version 1.26 in RMarkdown.

## Results

### Participants

A total of 502 540 people aged 40–69 years were recruited from 22 UK centres into the UK Biobank cohort study. Only 41 199 participants underwent an MRI scan at three assessment centres between 2014 and 2019, of whom 38 347 had WMH volume data available. Participants were not included if they had diagnoses that could confound WMH assessment (*N* = 492) or missing BP values at baseline and at follow-up (*N* = 17 and 822), resulting in 37 026 people included in the longitudinal analyses and 37 041 in cross-sectional analyses. Included participants were similar to the overall UK Biobank population but with more active smokers and fewer people with diabetes, angina, myocardial infarct, or antihypertensive medication (*Table 1*). The median time between the visits was 9.07 (range 4.28–13.49) years.

**Table 1:** Baseline characteristics of participants

Variable	Longitudinal—baseline	Longitudinal—follow-up	Longitudinal—difference	Cross-sectional
*N*	37 026	37 026		37 041
Age (years)	55.29 (7.45)	64.12 (7.52)	8.84 (8.82, 8.85)	64.12 (7.52)
Female sex, *n* (%)	19 624 (53.0)	.	.	19 635 (53.0)
Height (cm)	169.6 (9.16)	169.07 (9.24)	−0.54 (−0.56, −0.53)	169.07 (9.24)
Weight (kg)	76.57 (14.77)	75.93 (15.04)	−0.69 (−0.74, −0.63)	75.94 (15.06)
BMI (kg/m^2^)	26.53 (4.2)	26.47 (4.36)	−0.06 (−0.08, −0.04)	26.48 (4.37)
Diabetes, *n* (%)	908 (2.5)	.	.	1855 (5.1)
Ex-smoker, *n* (%)	12 057 (32.6)	12 332 (33.5)	275	12 334 (33.5)
Active smoker, *n* (%)	2278 (6.2)	1255 (3.4)	−1023	1256 (3.4)
SBP (mmHg)	134.81 (17.66)	138.26 (18.73)	3.45 (3.28, 3.62)	138.26 (18.72)
DBP (mmHg)	81.39 (9.92)	77.66 (10.65)	−3.72 (−3.83, −3.62)	77.66 (10.65)
PP (mmHg)	53.42 (12.29)	60.6 (15.16)	7.17 (7.04, 7.31)	60.59 (15.16)
ASI	9.07 (2.91)	9.61 (2.91)	0.48 (0.41, 0.55)	9.61 (2.91)
Myocardial infarct, *n* (%)	390 (1.3)	.	.	169 (2.4)
Angina, *n* (%)	560 (1.9)	.	.	202 (2.9)
Hypertension, *n* (%)	7089 (19.4)	.	.	2422 (26.4)
Antihypertensive medication, *n* (%)	4757 (14.6)	8264 (26.1)	3507	8269 (26.1)

Values are reported as mean (standard deviation) or as frequency (%).

ASI, arterial stiffness index; BMI, body mass index; DBP, diastolic blood pressure; SBP, systolic blood pressure; PP, pulse pressure.

### Relationship between concurrent and past blood pressure and white matter hyperintensities

WMH load was strongly associated with concurrent BP, both SBP and DBP, following adjustment for the other BP measure, age, sex, cardiovascular risk factors, source of BP measures, and the assessment centre (*Table 2*). A history of diabetes, smoking and increased arterial stiffness were also strongly associated with WMH. Although the effect of SBP diminished with adjustment, concurrent SBP remained a stronger predictor than concurrent DBP.

**Table 2:** Linear associations between white matter hyperintensities and concurrent blood pressure, age, sex, and cardiovascular risk factors

Variable	Unadjusted (95% CI)	Age and sex adjusted (95% CI)	Fully adjusted (95% CI)
SBP	0.273 (0.262 to 0.284)	0.126 (0.116 to 0.136)	0.076 (0.062 to 0.090)
DBP	0.083 (0.071 to 0.094)	0.117 (0.107 to 0.127)	0.064 (0.050 to 0.078)
ASI	0.073 (0.062 to 0.084)	0.032 (0.023 to 0.042)	0.011 (0.001 to 0.021)
Age	0.525 (0.515 to 0.534)		0.501 (0.490 to 0.511)
Female sex	−0.067 (−0.089 to −0.045)		0.083 (0.064 to 0.102)
Diabetes	0.452 (0.401 to 0.503)	0.309 (0.266 to 0.353)	0.301 (0.258 to 0.344)
Active smoker	0.055 (−0.006 to 0.116)	0.207 (0.154 to 0.259)	0.222 (0.171 to 0.274)
Ex-smoker	0.207 (0.184 to 0.231)	0.075 (0.054 to 0.095)	0.073 (0.053 to 0.093)

Associations are presented as standardized coefficients from general linear models. The median white matter hyperintensities volume was 2796 mm^3^ (range: 9 − 101 414), which corresponded to median 0.0052 (range: 0.000018 − 0.187) after normalizing by the total white matter volume.

ASI, arterial stiffness index; CI, confidence interval; DBP, diastolic blood pressure; SBP, systolic blood pressure.

In absolute terms, in fully adjusted models, there was a 1.041 [95% confidence interval (CI) 1.034–1.049] WMH load increase per 10 mmHg of concurrent SBP and 1.031 (95% CI 1.024–1.037) increase per 5 mmHg of concurrent DBP.

Given the greater population burden of elevated SBP even in midlife,^11^ the fully adjusted PAF of people in the top decile of WMH load was 19.1% for SBP over 120 mmHg and 6.8% for DBP over 70 mmHg.

In the longitudinal analyses, past values of SBP and DBP were positively associated with a higher WMH load later in life in unadjusted, age- and sex-adjusted, and fully adjusted models (*Table 3*). However, WMH load was more strongly associated with past DBP than concurrent DBP, with significant interaction with age (Supplementary material online, *Table S1*). In fully adjusted models, WMH load was also more strongly predicted by past DBP than by past SBP (*Table 3*). Again, diabetes and smoking at baseline were associated with increased WMH, but with little difference to the association with concurrent diabetes or smoking.

**Table 3:** Linear associations between white matter hyperintensities and past blood pressure, age, sex, and cardiovascular risk factors

Variable	Unadjusted (95% CI)	Age and sex adjusted (95% CI)	Fully adjusted (95% CI)
SBP	0.257 (0.240 to 0.274)	0.115 (0.100 to 0.131)	0.045 (0.022 to 0.069)
DBP	0.150 (0.133 to 0.167)	0.120 (0.105 to 0.136)	0.087 (0.064 to 0.109)
ASI	0.144 (0.126 to 0.161)	0.035 (0.019 to 0.051)	0.021 (0.005 to 0.037)
Age	0.494 (0.479 to 0.509)	.	0.480 (0.463 to 0.496)
Female sex	−0.046 (−0.080 to −0.011)	.	0.103 (0.072 to 0.133)
Diabetes	0.365 (0.259 to 0.472)	0.219 (0.127 to 0.311)	0.223 (0.133 to 0.313)
Active smoker	0.120 (0.045 to 0.194)	0.221 (0.156 to 0.286)	0.248 (0.185 to 0.311)
Ex-smoker	0.212 (0.174 to 0.249)	0.066 (0.033 to 0.099)	0.066 (0.034 to 0.098)
Time difference	0.131 (0.111 to 0.151)	0.163 (0.146 to 0.181)	0.183 (0.161 to 0.204)

Associations are presented as standardized coefficients from general linear models. The median white matter hyperintensities volume was 2796 mm^3^ (range: 9 − 101 414), which corresponded to median 0.0052 (range: 0.000018 − 0.187) after normalizing by the total white matter volume.

ASI, arterial stiffness index; CI, confidence interval; DBP, diastolic blood pressure; SBP, systolic blood pressure.

There was also a greater magnitude of association with DBP in the fully adjusted analysis with WMH load being 1.026 (95% CI 1.013–1.040) higher per 10 mmHg of past SBP and 1.045 (95% CI 1.033–1.057) higher per 5 mmHg of past DBP.

Overall, the fully adjusted PAF of people in the top decile of WMH load associated with past SBP over 120 mmHg was 24.4% and associated with past DBP over 70 mmHg was only 7.1%, reflecting the greater incidence of systolic and mixed HT in the population.

### Effect of stratification by age, sex, and duration of follow-up

The association between concurrent BP and WMH load was greater for SBP than for DBP across different age groups, especially under the age of 70. In contrast, the pattern of association between past BP and WMH varied according to age decade (Supplementary material online, *Figure S1*; standardized effects: Supplementary material online, *Tables S2* and *S3*; non-standardized effects: Supplementary material online, *Table S4*). The patterns of association were comparable for men and women both for concurrent (Supplementary material online, *Figure S2*) and past BP (Supplementary material online, *Figure S3*).

The greater effect of past DBP in younger participants was even more pronounced with short follow-up time (Supplementary material online, *Figure S4*). Nonetheless, the PAF for severe WMH was greater for SBP, both concurrent and past, except for the youngest age group, due to the higher prevalence of systolic HT with increasing age (Supplementary material online, *Table S5*).

### Relationship with usual blood pressure

**Figure 1:**
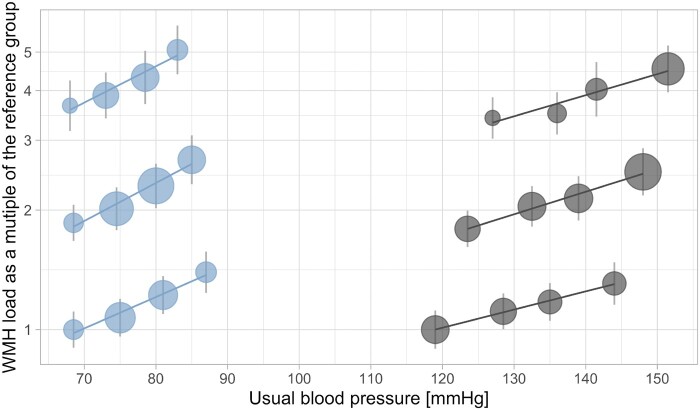
Increase in median white matter hyperintensities across usual blood pressure groups, stratified by age. Median white matter hyperintensities are presented on a logarithmic scale as a ratio of median white matter hyperintensities per patient group compared to the reference group (age < 50 years and systolic blood pressure <120 mmHg or diastolic blood pressure <70 mmHg), stratified by age (<50, 50–60, ≥60 years) and blood pressure at baseline (diastolic blood pressure <70, 70–80, 80–90, ≥90 mmHg; systolic blood pressure <120, 120–130, 130–140, ≥140 mmHg). Diastolic blood pressure plotted in blue on the left and systolic blood pressure plotted in grey on the right. Lines represent a linear fit across all points within age group on a logarithmic scale.

Following adjustment for regression dilution bias, there was a consistent log-linear relationship between concurrent SBP and DBP and severity of WMH in each age group (*Figure 1*), including patients with BP below 140 mmHg for systolic and below 90 mmHg for diastolic. Overall, there was a 1.126 (95% CI 1.107–1.146) increase in median WMH load per 10 mmHg SBP and a 1.106 (95% CI 1.090–1.122) increase per 5 mmHg DBP. This was consistent in the youngest decade, 1.111 (95% CI 1.092–1.131) increase per 10 mmHg usual SBP and 1.095 (95% CI 1.047–1.145) increase per 5 mmHg DBP, and the oldest age group, 1.130 (95% CI 1.011–1.263) increase per 10 mmHg usual SBP and 1.111 (95% CI 1.030–1.199) increase per 5 mmHg DBP.

Across most age groups, the relationship consistently increased across BP levels, with a rise in WMH even in normotensive patients (SBP <130 mmHg or DBP <80 mmHg), with no overall evidence of a j-shaped curve (*Figure 1*). However, in the eldest participants, there was no difference between median WMH in the group with SBP <130 mmHg.

### Relationship between white matter hyperintensities, hypertension, and antihypertensive medication

**Figure 2:**
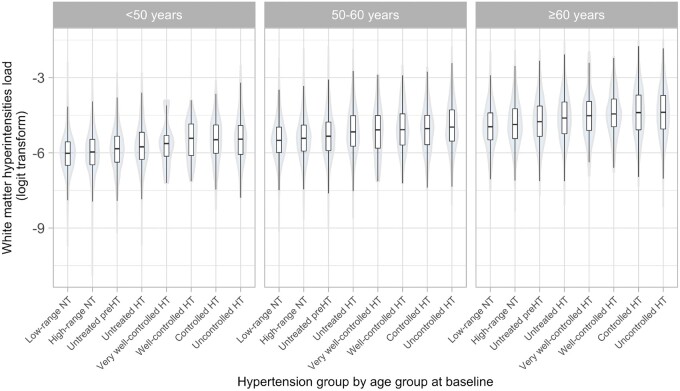
Median white matter hyperintensities load, stratified by age, definition of hypertension, and treatment at baseline. HT, hypertensive; NT, normotensive.

Higher WMH load was associated with higher past BP and antihypertensive treatment across all recorded BP levels and all age groups (*Figure 2* and Supplementary material online, *Table S6*). Higher WMH load in people on antihypertensive medication was present even if BP values were within the normotensive or pre-hypertensive range. A similar pattern of associations was present in the cross-sectional analysis (Supplementary material online, *Figure S5* and Supplementary material online, *Table S7*).

## Discussion

In UK Biobank, WMH were strongly associated with both concurrent and past BP, as well as diabetes and smoking, before and after adjustment for age, sex, and other cardiovascular risk factors. WMH were more strongly associated with past DBP than with past SBP, especially under the age of 50. Nonetheless, associations between usual BP and median WMH load were log-linear in all age groups for both SBP and DBP, although there was a possible ‘j-shaped’ relationship in the eldest age group. Despite the stronger direct association with past DBP, the higher prevalence of systolic HT meant that there was a greater population attributable risk for severe WMH due to SBP than DBP.

Previous observational studies reported associations for concurrent SBP with WMH, independent of age, sex, and cardiovascular risk factors,^9^ ^,^ ^13^ ^,^ ^22^ ^,^ ^23^ except for one computer tomography-based study identifying a stronger association with DBP.^24^ These studies mostly imaged older participants (>65 years)^13^ ^,^ ^22^ ^,^ ^24^ with higher baseline BP levels.^22^ In contrast, in longitudinal studies, late-life WMH have been associated with midlife BP, with differences in the strength of association for DBP^12–15^ ^,^ ^22^ ^,^ ^24^ vs. SBP.^12^ ^,^ ^13^ ^,^ ^22^ Due to the lack of imaging studies in younger participants and variation in exposure periods, these studies did not determine the key exposure period for elevated BP that is crucial for the development of WMH. Given the size and age distribution of the UK Biobank cohort, this study demonstrated that, in early midlife (<50 years), past DBP was more important for the development of WMH later in life. Furthermore, the population was sufficiently large to demonstrate an increased association for past BP even within the pre-hypertensive range. Greater WMH in patients with a diagnosis of HT, independent on measured BP level, also supports the conjecture that long-term high BP exposure, rather than transient raised BP, is critical for WMH development.

There was no evidence for an interaction between time of exposure for diabetes and smoking, and randomized studies have not sufficiently assessed the effect of smoking and diabetes control on WMH prevention. In contrast, there was a time-dependent interaction between BP and future WMH. This implies a potential pathophysiological mechanism and is in line with Trails demonstrating that intensive BP lowering within the pre-hypertensive range is associated with a reduction in the progression of WMH in patients with prior stroke^25^ or increased cardiovascular risk.^10^ ^,^ ^26^ Our results suggest that, to ensure maximal prevention of WMH in late life, control of DBP may be required in early midlife, even for DBP below 90 mmHg, whilst control of SBP may be more important in late life. However, current guidelines for the initiation of antihypertensive treatment depend upon a global cardiovascular risk assessment, which is strongly influenced by age and derived from cardiovascular events in medium-term clinical trials. Further evidence is, therefore, required to assess the potential long-term benefits of more intensive BP control at younger ages, ideally through long-term, large trials but, more practically, from longer-term follow-up of previously established trials and large cohorts. Further analysis of UK Biobank may demonstrate associations with specific drug classes, whilst the lower WMH in treated patients with lower BP could imply an effect of treatment. However, this is confounded by the indication for treatment, as demonstrated by the systematically greater WMH for patients on antihypertensives reflecting long-term BP exposure regardless of acutely measured BP level.

The strengths of this study include the large sample of population-based participants, a prospective design allowing adjustment for regression dilution bias, fully automated quantitative MRI data, and prospective follow-up. However, there were some limitations. First, large-sample MRI is currently only available at one time point and, therefore, the progression of WMH could not be directly quantified compared to other studies.^24^ ^,^ ^27^ However, the very large sample size allows for detailed stratification of the population by age as a surrogate for progression within the population. In future, repeated MRI within UK Biobank will enable the quantification of progression of MRI indices. Second, the quantitative MRI data combine both deep and periventricular WMH, which may have different pathophysiological relationships with the longitudinal evolution of BP. Additional analysis of UK Biobank images will be required to identify pathophysiological differences in different regions of white matter. Third, as further data are acquired by UK Biobank and sequentially released, this will include primary care treatment records for all participants, allowing the determination of drug class-specific effects and the effects of treatment initiation on BP control and WMH. Fourth, the interval between baseline assessment and MRI varied significantly, requiring adjustment for the time difference in the longitudinal models, assuming a linear rate of progression of WMH. This limits the use of external populations to derive the usual BP transformations. Therefore, the usual BP was derived from the less-biased follow-up BP values of the subgroups being investigated in this study. Moreover, as only clinic readings were available, masked, or white coat HT may result in a non-representative allocation of patients to BP strata (mainly the untreated pre-hypertensive and untreated hypertensive), affecting the association between BP and WMH. Finally, this analysis focused on the interaction between BP as the key modifiable risk factor for WMH to inform current treatment strategies. Although we also demonstrated associations with smoking and diabetes, the complex potential interactions between other modifiable risk factors such as smoking, diabetes, dyslipidaemia, obesity, and renal dysfunction were beyond the scope of this analysis and will require further dedicated studies.

## Conclusions

Increased BP was associated with higher WMH load, with particularly strong associations between increased WMH and DBP before the age of 50 and SBP after the age of 50, even at BP values below common treatment thresholds. Understanding the associations between BP and WMH, especially in younger people at a pre-hypertensive stage, may help to identify the optimal approach to preserve HT-associated, long-term vascular health.

## Supplementary material

Supplementary material is available at *European Heart Journal* online.

## Data availability statement

This study used UK Biobank data that are available to eligible researchers after registration.

## Authors’ contributions

K.A.W. designed and performed analysis of the data and wrote the manuscript. A.J.S.W. initiated, designed, and supervised the study and its analysis and wrote the manuscript. Both authors read and approved the final manuscript. 

## Authors’ status

K.A.W.: Clinical Research Fellow, Wolfson Centre for Prevention of Stroke and Dementia, Nuffield Department of Clinical Neurosciences, University of Oxford; A.J.S.W.: Wellcome Trust Clinical Research Development Fellow/Honorary Neurology Consultant, Wolfson Centre for Prevention of Stroke and Dementia, Nuffield Department of Clinical Neurosciences, University of Oxford.

## Funding

This work was supported by an Alzheimer’s Society grant (450-AS-PG-18-018). A.J.S.W. is also funded by a Wellcome Trust Clinical Research Career Development Fellowship (206589/Z/17/Z). The funders of the study had no role in study design, data collection, data analysis, data interpretation, or writing of the manuscript. The corresponding author had full access to all the data in the study and had final responsibility for the decision to submit for publication. 

**Conflict of interest:** none declared. 

## Supplementary Material

ehaa756_Supplementary_Data
