# Supplementary material for: Midlife blood pressure is associated with the severity of white matter hyperintensities: analysis of the UK Biobank cohort study
Source: Eur Heart J. 2020 Nov 26;42(7):750–7. doi: 10.1093/eurheartj/ehaa756 (PMC7882359; doi:10.1093/eurheartj/ehaa756)
Supplement: ehaa756_Supplementary_Data [file ehaa756_supplementary_data.docx]

# Supplementary data

#### Interactions between age and blood pressure in the fully-adjusted models

Supplementary Table 1: **Interactions between the effect of age and blood pressure on white matter hyperintensity in a fully-adjusted cross-sectional (left) and longitudinal (right) analysis.** The values represent standardised coefficients and their 95% confidence intervals.

| **Variable** | **Cross-sectional** | **Longitudinal** |
| --- | --- | --- |
| SBP | 0.046 (0.026 to 0.067) | -0.026 (-0.063 to 0.010) |
| DBP | 0.079 (0.059 to 0.099) | 0.113 (0.080 to 0.146) |
| Age | 0.503 (0.492 to 0.514) | 0.482 (0.465 to 0.498) |
| Age*SBP | -0.018 (-0.031 to -0.005) | 0.049 (0.027 to 0.072) |
| Age*DBP | 0.112 (-0.133 to 0.356) | -0.025 (-0.047 to -0.004) |
| Female sex | 0.085 (0.066 to 0.105) | 0.092 (0.061 to 0.123) |
| Female sex*SBP | -0.023 (-0.049 to 0.002) | 0.088 (0.045 to 0.132) |
| Female sex*DBP | 0.009 (-0.004 to 0.022) | -0.019 (-0.062 to 0.024) |

##### page break

#### Sensitivity analyses with WMH as a fraction of total brain volume

Supplementary Table 2: **Linear associations between white matter hyperintensities normalised by the total brain volume and concurrent blood pressure, age, sex, and cardiovascular risk factors at follow-up.** The values represent standardised coefficients and their 95% confidence intervals.

| Variable | Unadjusted (95%CIs) | Age- and sex-adjusted (95%CIs) | Fully-adjusted (95%CIs) |
| --- | --- | --- | --- |
| SBP | 0.275 (0.264 to 0.286) | 0.126 (0.116 to 0.136) | 0.076 (0.062 to 0.089) |
| DBP | 0.085 (0.073 to 0.097) | 0.117 (0.107 to 0.128) | 0.065 (0.051 to 0.079) |
| ASI | 0.075 (0.064 to 0.086) | 0.033 (0.023 to 0.042) | 0.011 (0.002 to 0.021) |
| Age | 0.525 (0.516 to 0.535) | . | 0.500 (0.490 to 0.511) |
| Female sex | -0.087 (-0.109 to -0.064) | . | 0.064 (0.045 to 0.084) |
| Diabetes | 0.458 (0.407 to 0.508) | 0.312 (0.268 to 0.355) | 0.303 (0.260 to 0.346) |
| Active smoker | 0.060 (0.000 to 0.121) | 0.210 (0.158 to 0.262) | 0.225 (0.174 to 0.276) |
| Ex-smoker | 0.211 (0.187 to 0.234) | 0.077 (0.057 to 0.098) | 0.075 (0.055 to 0.095) |
|  |  |  |  |

Supplementary Table 3: **Linear associations between white matter hyperintensities normalised by the total brain volume and past blood pressure, age, sex, and cardiovascular risk factors.** The values represent standardised coefficients and their 95% confidence intervals.

| Variable | Unadjusted (95%CIs) | Age- and sex-adjusted (95%CIs) | Fully-adjusted (95%CIs) |
| --- | --- | --- | --- |
| SBP | 0.260 (0.243 to 0.277) | 0.116 (0.100 to 0.132) | 0.046 (0.022 to 0.069) |
| DBP | 0.153 (0.136 to 0.170) | 0.121 (0.106 to 0.136) | 0.087 (0.065 to 0.110) |
| ASI | 0.147 (0.129 to 0.164) | 0.036 (0.020 to 0.052) | 0.021 (0.006 to 0.037) |
| Age | 0.496 (0.481 to 0.511) | . | 0.480 (0.463 to 0.496) |
| Female sex | -0.066 (-0.100 to -0.031) | . | 0.084 (0.053 to 0.114) |
| Diabetes | 0.373 (0.267 to 0.479) | 0.223 (0.131 to 0.315) | 0.227 (0.138 to 0.317) |
| Active smoker | 0.126 (0.052 to 0.201) | 0.225 (0.160 to 0.290) | 0.252 (0.189 to 0.315) |
| Ex-smoker | 0.215 (0.177 to 0.252) | 0.068 (0.035 to 0.101) | 0.068 (0.036 to 0.100) |
| Time difference | 0.125 (0.105 to 0.145) | 0.158 (0.140 to 0.175) | 0.177 (0.156 to 0.198) |

##### page break

#### Sensitivity analyses with full model adjusted for antihypertensive medication

Supplementary Table 4: **Results of cross-sectional fully-adjusted model with antihypertensive medication.**

| Variable | Unadjusted (95%CIs) | Age- and sex-adjusted (95%CIs) | Fully-adjusted (95%CIs) |
| --- | --- | --- | --- |
| SBP | 0.273 (0.262 to 0.284) | 0.126 (0.116 to 0.136) | 0.066 (0.051 to 0.081) |
| DBP | 0.083 (0.071 to 0.095) | 0.117 (0.107 to 0.127) | 0.060 (0.046 to 0.075) |
| ASI | 0.073 (0.062 to 0.085) | 0.032 (0.023 to 0.042) | 0.013 (0.002 to 0.023) |
| Age | 0.525 (0.515 to 0.534) | . | 0.477 (0.466 to 0.489) |
| Female sex | -0.067 (-0.090 to -0.045) | . | 0.091 (0.070 to 0.111) |
| Diabetes | 0.452 (0.401 to 0.503) | 0.309 (0.266 to 0.353) | 0.206 (0.155 to 0.257) |
| Active smoker | 0.055 (-0.005 to 0.116) | 0.206 (0.154 to 0.259) | 0.200 (0.145 to 0.256) |
| Ex-smoker | 0.207 (0.184 to 0.231) | 0.075 (0.055 to 0.095) | 0.056 (0.034 to 0.077) |
| Anti-hypertensives | 0.593 (0.567 to 0.619) | 0.306 (0.282 to 0.331) | 0.247 (0.222 to 0.271) |

Supplementary Table 5: **Results of longitudinal fully-adjusted model with antihypertensive medication**

| Variable | Unadjusted (95%CIs) | Age- and sex-adjusted (95%CIs) | Fully-adjusted (95%CIs) |
| --- | --- | --- | --- |
| SBP | 0.257 (0.240 to 0.274) | 0.115 (0.100 to 0.131) | 0.035 (0.011 to 0.060) |
| DBP | 0.150 (0.133 to 0.167) | 0.120 (0.105 to 0.136) | 0.081 (0.058 to 0.105) |
| ASI | 0.144 (0.126 to 0.161) | 0.035 (0.019 to 0.051) | 0.017 (0.000 to 0.033) |
| Age | 0.494 (0.479 to 0.509) | . | 0.462 (0.445 to 0.480) |
| Female sex | -0.046 (-0.080 to -0.011) | . | 0.110 (0.078 to 0.143) |
| Diabetes | 0.365 (0.259 to 0.472) | 0.219 (0.127 to 0.311) | 0.107 (-0.004 to 0.218) |
| Active smoker | 0.120 (0.045 to 0.194) | 0.221 (0.156 to 0.286) | 0.245 (0.178 to 0.312) |
| Ex-smoker | 0.212 (0.174 to 0.249) | 0.066 (0.033 to 0.099) | 0.053 (0.019 to 0.087) |
| Anti-hypertensives | 0.622 (0.573 to 0.672) | 0.320 (0.275 to 0.365) | 0.269 (0.223 to 0.315) |
| Time difference | 0.131 (0.111 to 0.151) | 0.163 (0.146 to 0.181) | 0.107 (0.094 to 0.120) |

##### page break

#### Sensitivity analyses with full model using automated BP only and automated BP

Supplementary Table 6: **Results of cross-sectional fully-adjusted model with automated BP.**

| Variable | Unadjusted (95%CIs) | Age- and sex-adjusted (95%CIs) | Fully-adjusted (95%CIs) |
| --- | --- | --- | --- |
| SBP | 0.275 (0.264 to 0.287) | 0.126 (0.115 to 0.136) | 0.075 (0.060 to 0.089) |
| DBP | 0.085 (0.073 to 0.098) | 0.119 (0.109 to 0.130) | 0.067 (0.053 to 0.082) |
| ASI | 0.072 (0.060 to 0.084) | 0.032 (0.022 to 0.042) | 0.011 (0.001 to 0.021) |
| Age | 0.529 (0.519 to 0.539) | . | 0.505 (0.494 to 0.516) |
| Female sex | -0.071 (-0.095 to -0.048) | . | 0.080 (0.060 to 0.100) |
| Diabetes | 0.452 (0.400 to 0.505) | 0.310 (0.265 to 0.355) | 0.302 (0.257 to 0.346) |
| Active smoker | 0.026 (-0.037 to 0.090) | 0.178 (0.124 to 0.233) | 0.194 (0.140 to 0.248) |
| Ex-smoker | 0.204 (0.179 to 0.228) | 0.072 (0.051 to 0.093) | 0.070 (0.049 to 0.091) |

Supplementary Table 7: **Results of longitudinal fully-adjusted model with automated BP**

| Variable | Unadjusted (95%CIs) | Age- and sex-adjusted (95%CIs) | Fully-adjusted (95%CIs) |
| --- | --- | --- | --- |
| SBP | 0.258 (0.241 to 0.275) | 0.116 (0.100 to 0.132) | 0.046 (0.023 to 0.069) |
| DBP | 0.151 (0.134 to 0.169) | 0.121 (0.106 to 0.137) | 0.087 (0.065 to 0.110) |
| ASI | 0.144 (0.127 to 0.161) | 0.036 (0.020 to 0.052) | 0.021 (0.006 to 0.037) |
| Age | 0.494 (0.479 to 0.509) | . | 0.479 (0.462 to 0.495) |
| Female sex | -0.046 (-0.081 to -0.011) | . | 0.103 (0.072 to 0.134) |
| Diabetes | 0.366 (0.260 to 0.472) | 0.220 (0.127 to 0.312) | 0.224 (0.134 to 0.314) |
| Active smoker | 0.120 (0.046 to 0.195) | 0.221 (0.156 to 0.286) | 0.248 (0.185 to 0.311) |
| Ex-smoker | 0.210 (0.172 to 0.247) | 0.065 (0.032 to 0.098) | 0.065 (0.032 to 0.097) |
| Time difference | 0.130 (0.110 to 0.150) | 0.163 (0.145 to 0.180) | 0.106 (0.093 to 0.118) |

##### page break

#### Effect of SBP and DBP in cross-sectional and longitudinal analyses stratified by age group


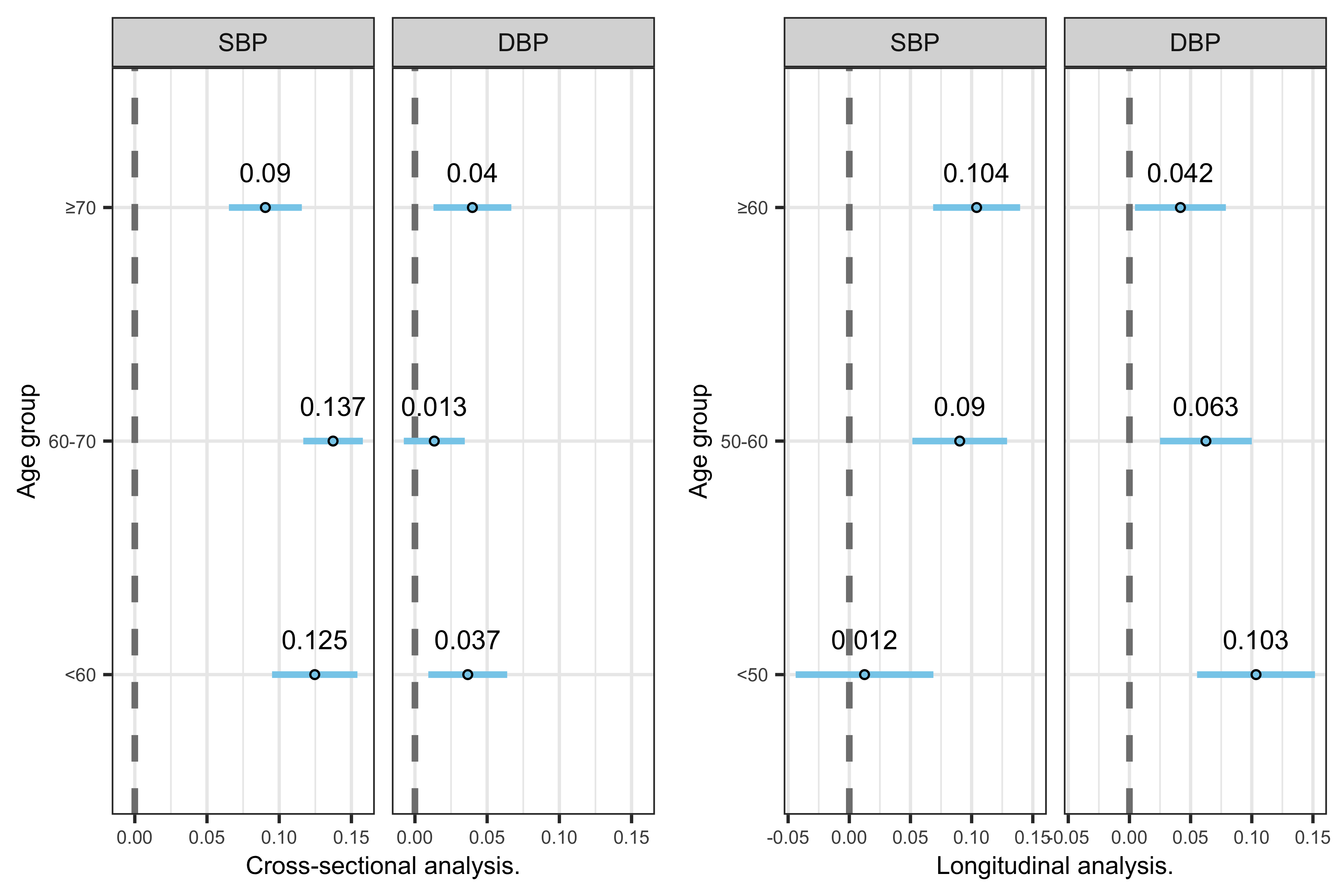
 Supplementary Figure 1: **Cross-sectional (left) and longitudinal (right) analysis stratified per age group.** The values represent standardised coefficients and their 95% confidence intervals.

Supplementary Table 8: **Cross-sectional analyses stratified by age group.** The table corresponds to the left-hand side forest plot in Supplementary Figure 1. The values represent standardised coefficients and their 95% confidence intervals.

| Group | BP | Fully-adjusted |
| --- | --- | --- |
| <60 | SBP | 0.125 (0.095 to 0.154) |
| 60-70 | SBP | 0.137 (0.117 to 0.158) |
| ≥70 | SBP | 0.090 (0.065 to 0.116) |
| <60 | DBP | 0.037 (0.009 to 0.064) |
| 60-70 | DBP | 0.013 (-0.008 to 0.035) |
| ≥70 | DBP | 0.040 (0.013 to 0.067) |

Supplementary Table 9: **Longitudinal analyses stratified by age group.** The table corresponds to the right-hand forest plot in Supplementary Figure 1. The values represent standardised coefficients and their 95% confidence intervals.

| Group | BP | Fully-adjusted |
| --- | --- | --- |
| <50 | SBP | 0.012 (-0.044 to 0.069) |
| 50-60 | SBP | 0.090 (0.052 to 0.129) |
| ≥60 | SBP | 0.104 (0.069 to 0.140) |
| <50 | DBP | 0.103 (0.055 to 0.152) |
| 50-60 | DBP | 0.063 (0.025 to 0.100) |
| ≥60 | DBP | 0.042 (0.005 to 0.079) |

Supplementary Table 10: **Results of cross-sectional and longitudinal analyses by age group**. Increase in WMH per 10 mmHg SBP or 5 mmHg DBP.

| **Age group** | **WMH increase per 10 mmHg concurrent SBP** | **WMH increase per 5 mmHg concurrent DBP** |
| --- | --- | --- |
| < 60 | 1.069 | 1.017 |
| 60 - 70 | 1.076 | 1.0063 |
| ≥ 70 | 1.049 | 1.019 |
| **Age group** | **WMH increase per 10 mmHg past SBP** | **WMH increase per 5 mmHg past DBP** |
| < 50 | 1.007 | 1.054 |
| 50 - 60 | 1.052 | 1.032 |
| ≥ 60 | 1.061 | 1.021 |

##### page break

#### Effect of SBP and DBP in cross-sectional and longitudinal analyses stratified by sex


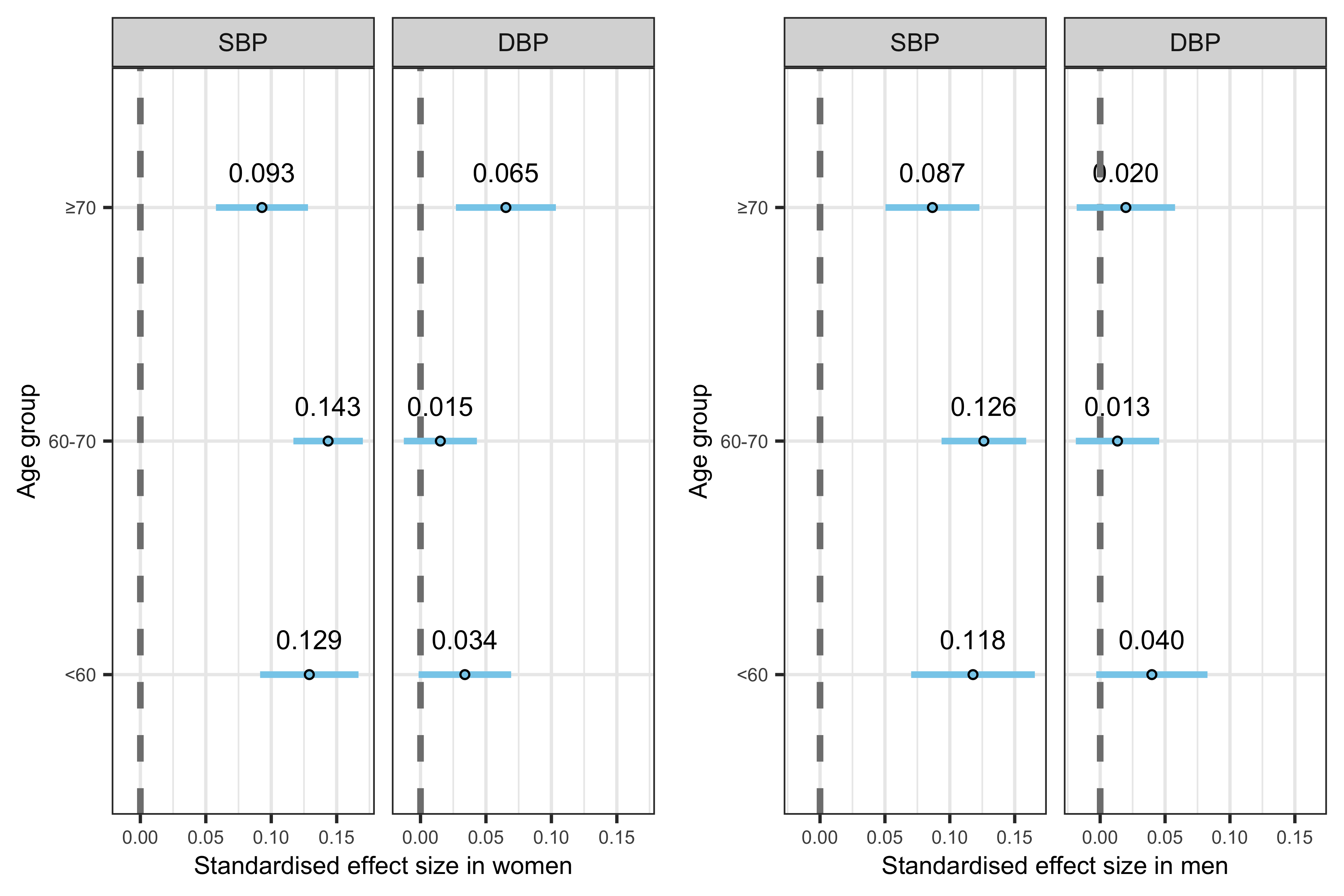


Supplementary Figure 2: **Cross-sectional analyses stratified by age in women (left) and in men (right).** The values represent standardised coefficients and their 95% confidence intervals.


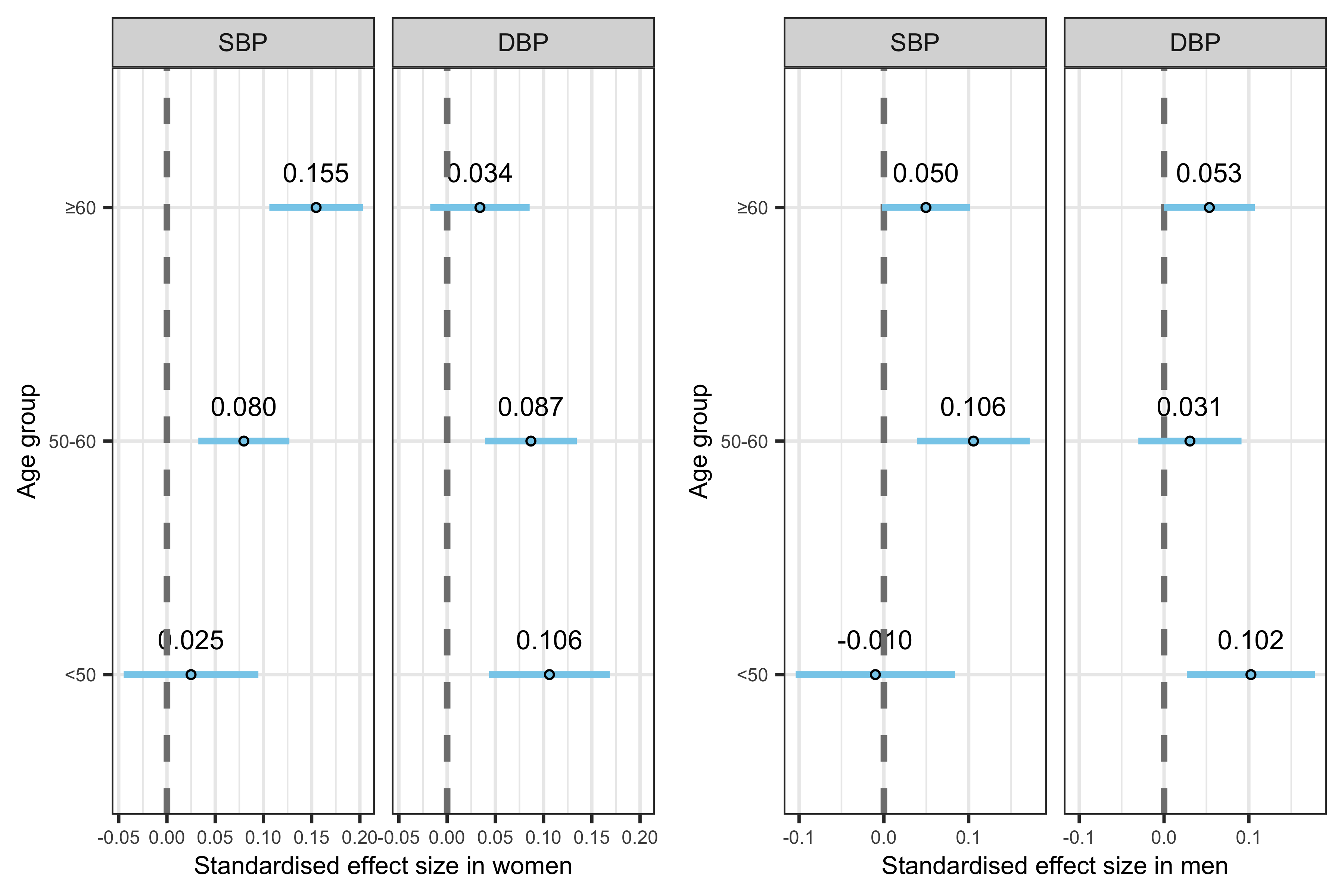
 Supplementary Figure 3: **Longitudinal analyses stratified by age in women (left) and men (right).** The values represent standardised coefficients and their 95% confidence intervals from a fully-adjusted model.

##### page break

#### Effect of SBP and DBP in cross-sectional and longitudinal analyses stratified by age group and follow-up duration


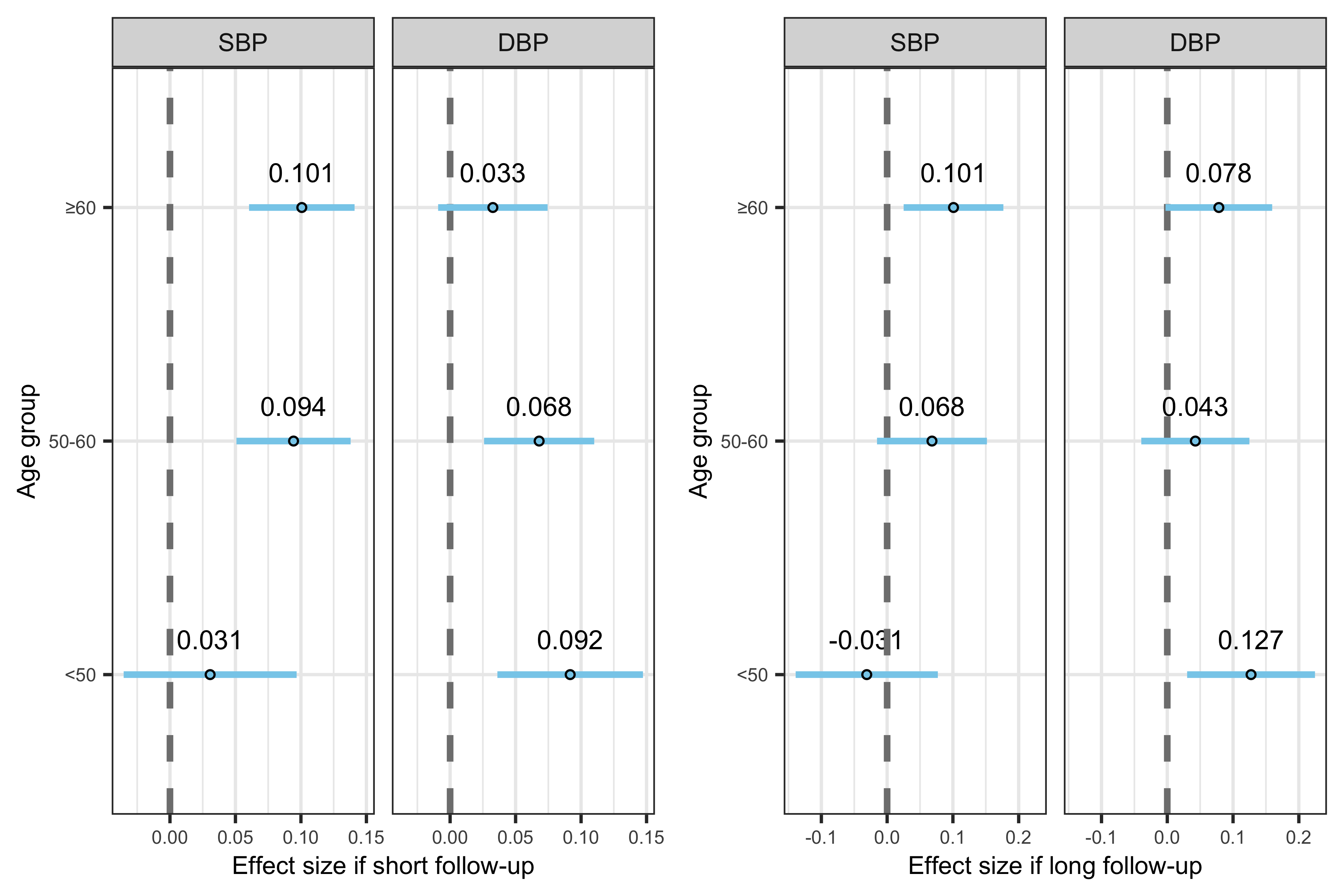
 Supplementary Figure 4: **Longitudinal analysis stratified by age and duration of follow-up.** The values represent standardised coefficients and their 95% confidence intervals from a fully-adjusted model.

##### page break

#### Population attributable fraction stratified by age group

Supplementary Table 11: **Population attributable fraction (PAF) of the population by age group in the cross-sectional and longitudinal analysis per age group.** The proportion of people in the highest decile of WMH attributable to SBP>120 mmHg or DBP>70 mmHg.

| **Age group** | **WMH PAF for concurrent SBP over 120mmHg** | **WMH PAF for concurrent DBP over 70mmHg** |
| --- | --- | --- |
| < 60 | 11.6 | 17.7 |
| 60 - 69 | 30.1 | 10.6 |
| ≥ 70 | 18.4 | -0.8 |
| **Age group** | **WMH PAF for past SBP over 120mmHg** | **WMH PAF for past DBP over 70 mmHg** |
| < 50 | 5.3 | 10.7 |
| 50 - 59 | 20.2 | 5.6 |
| ≥ 60 | 41.2 | 23.1 |

##### page break

#### Interactions between WMH, hypertension, and antihypertensive medication


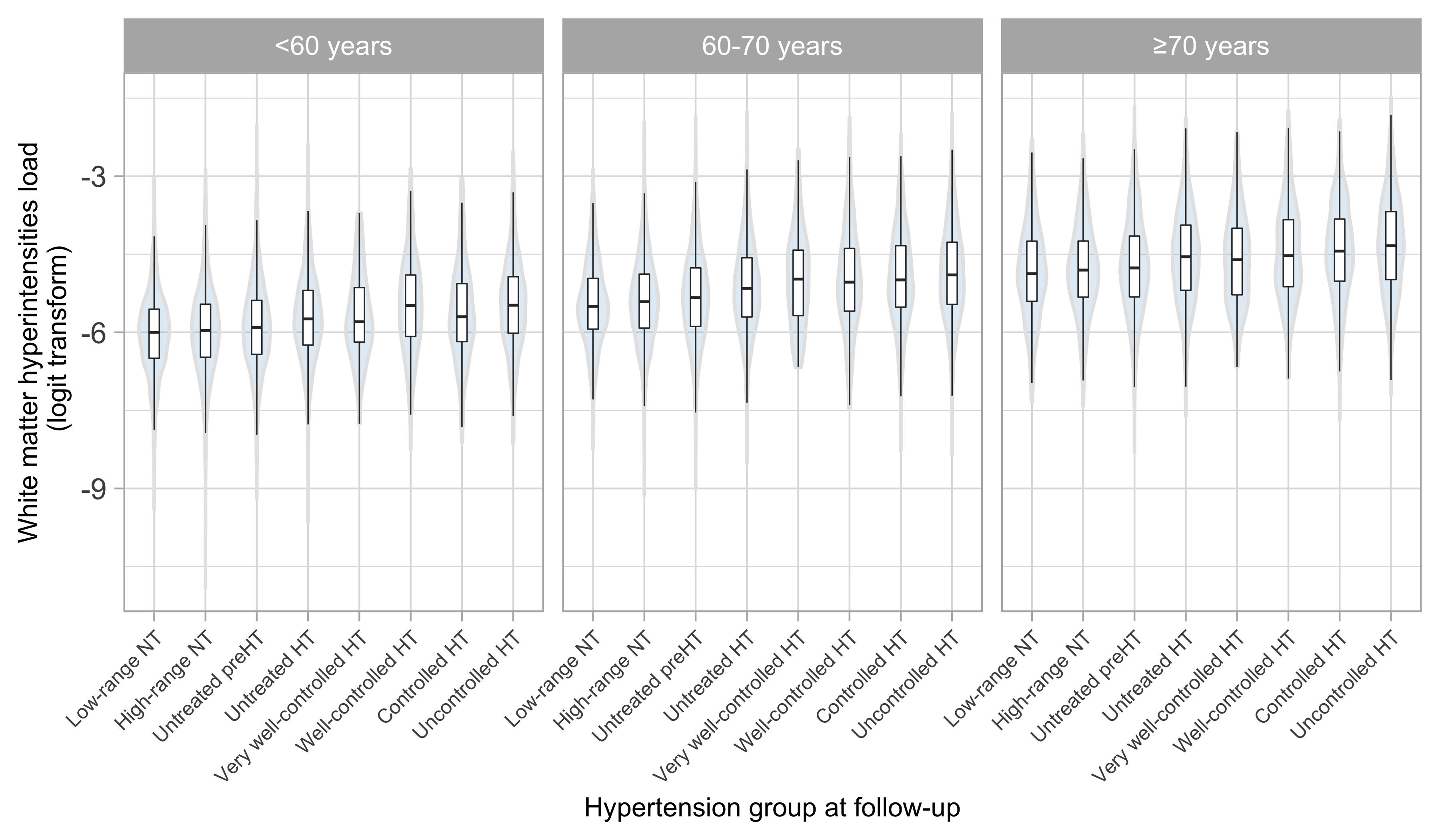


Supplementary Figure 5: **WMH stratified by age, hypertension, and antihypertensive medication status at follow-up.** NT – normotensive, HT – hypertensive.

Supplementary Table 12: **Increase in WMH in relation to people not on antihypertensive medication and with blood pressure below <120/70 mmHg at baseline.** NT – normotensive, HT – hypertensive.

| **Group at baseline** | **BP [mmHg]** | **AntiHT** | **Increase in WMH load (95% CIs)** | **N** |
| --- | --- | --- | --- | --- |
| High NT | SBP: 120-130 or DBP: 70-80 | No | 1.089 (1.018 to 1.166) | 7384 |
| Untreated pre-HT | SBP: 130-140 or DBP: 80-90 | No | 1.213 (1.135 to 1.298) | 8133 |
| Untreated HT | SBP ≥ 140 or DBP ≥ 90 | No | 1.415 (1.323 to 1.513) | 9780 |
| Very well-controlled HT | SBP < 120 or DBP: < 70 | Yes | 1.417 (1.078 to 1.863) | 122 |
| Well-controlled HT | SBP 120-130 or DBP 70-80 | Yes | 1.720 (1.496 to 1.978) | 491 |
| Controlled HT | SBP: 130-140 or 80-90 | Yes | 1.782 (1.606 to 1.976) | 1185 |
| Uncontrolled HT | ≥ 140 or DBP ≥ 90 | Yes | 1.791 (1.654 to 1.941) | 2959 |

Supplementary Table 13: **Increase in WMH in relation to people not on antihypertensive medication and with blood pressure < 120/70 mmHg at follow-up.** NT – normotensive, HT – hypertensive.

| **Group at follow-up** | **BP [mmHg]** | **AntiHT** | **Increase in WMH load (95% CIs)** | **N** |  |
| --- | --- | --- | --- | --- | --- |
| High NT | SBP: 120-130 or DBP: 70-80 | No | 1.079 (1.032 to 1.127) | 5463 |  |
| Untreated pre-HT | SBP: 130-140 or DBP: 80-90 | No | 1.180 (1.130 to 1.232) | 6053 |  |
| Untreated HT | SBP ≥ 140 or DBP ≥ 90 | No | 1.398 (1.341 to 1.457) | 9226 |  |
| Very well-controlled HT | SBP < 120 or DBP: < 70 | Yes | 1.545 (1.374 to 1.738) | 285 |  |
| Well-controlled HT | SBP 120-130 or DBP 70-80 | Yes | 1.603 (1.495 to 1.719) | 949 |  |
| Controlled HT | SBP: 130-140 or 80-90 | Yes | 1.604 (1.516 to 1.697) | 1879 |  |
| Uncontrolled HT | ≥ 140 or DBP ≥ 90 | Yes | 1.774 (1.693 to 1.858) | 5156 |  |

##### page break
